# iNOS Activity Is Required for the Therapeutic Effect of Mesenchymal Stem Cells in Experimental Systemic Sclerosis

**DOI:** 10.3389/fimmu.2018.03056

**Published:** 2018-12-21

**Authors:** Alexandre T. J. Maria, Pauline Rozier, Guillaume Fonteneau, Thibault Sutra, Marie Maumus, Karine Toupet, Jean-Paul Cristol, Christian Jorgensen, Philippe Guilpain, Danièle Noël

**Affiliations:** ^1^IRMB, University Montpellier, INSERM, CHU Montpellier, Montpellier, France; ^2^Department of Internal Medicine–Multi-organic Diseases, Saint-Eloi Hospital, Montpellier, France; ^3^Laboratory of Biochemistry, University Hospital of Montpellier, Montpellier, France; ^4^Inserm U1046, Phymedexp, Lapeyronie Hospital, Montpellier, France; ^5^Clinical Immunology and Osteoarticular Diseases Therapeutic Unit, Lapeyronie Hospital, Montpellier, France

**Keywords:** systemic sclerosis, HOCl, mesenchymal stem cells, inducible NO synthase, oxidative stress

## Abstract

**Objectives:** Fibrosis is a hallmark of systemic sclerosis (SSc), an intractable disease where innovative strategies are still being sought. Among novel anti-fibrotic approaches, mesenchymal stromal/stem cell (MSC)-based therapy appears promising. Previously, we reported anti-fibrotic effects of MSC in an experimental model of SSc, through various mechanisms (tissue remodeling, immunomodulation, anti-oxidant defense). Since immunomodulation is a pivotal mechanism for MSC therapeutic effects, we investigated the specific role of critical molecules associated with MSC immunosuppressive properties and hypothesized that MSC defective for these molecules would be less effective in reducing fibrosis in SSc.

**Methods:** SSc was induced by 6-week daily intradermal injections of hypochlorite (HOCl) in mice. MSC were isolated from the bone marrow of wild type mice (WT) or mice knockout for IL1RA, IL6, or iNOS (IL1RA^−/−^, IL6^−/−^, or iNOS^−/−^ MSC, respectively). Treated-mice received 2.5 × 10^5^ MSC intravenous infusion at d21. Skin thickness, histological and biological parameters were evaluated in skin and blood at d42.

**Results:** IL1RA^−/−^ and IL6^−/−^ MSC exerted similar anti-fibrotic properties as WT MSC, with a reduction of skin thickness together with less collagen deposition. Conversely, iNOS^−/−^ MSC did not exert anti-fibrotic functions as shown by a similar skin thickness progression as non-treated HOCl-SSc mice. Compared with WT MSC, iNOS^−/−^ MSC kept some immunosuppressive and tissue remodeling properties, but lost their capacity to reduce oxidative stress in HOCl-SSc mice.

**Conclusion:** Our study highlights the crucial role of iNOS, whose activity is required for the anti-fibrotic properties of MSC in experimental SSc, with a special emphasis on NO-related anti-oxidant functions.

## Introduction

Skin fibrosis is the hallmark of systemic sclerosis (SSc), a rare and intractable autoimmune disease characterized by multi-organ fibrosis where innovative therapeutic strategies are still being sought. Among novel anti-fibrotic approaches in development, mesenchymal stromal/stem cell (MSC)-based therapy appears promising (1). Our group previously reported dramatic anti-fibrotic and anti-inflammatory effects of MSC in an experimental mouse model of SSc (2, 3). This inducible model, based on daily exposure to hypochlorite (HOCl-SSc), mimics the main features of human SSc in its diffuse and rapidly progressive form. MSC immunosuppressive properties are pivotal for their therapeutic effects, and mainly rely on paracrine mechanisms depending on soluble factors secretion (4–6).

We previously demonstrated that MSC efficacy in HOCl-SSc was associated with huge decrease in tissue inflammation characterized by less T-lymphocytes and macrophages infiltrates, and lower levels of inflammatory cytokines (7). In the present study, we further investigated the specific role of some critical molecules associated with MSC immunosuppressive properties and hypothesized that MSC defective for interleukine-1 receptor-antagonist (IL1RA), interleukine-6 (IL6) or inducible nitric-oxide (NO)-synthase (iNOS) would be less effective in reducing fibrosis in SSc. Herein, through a concise report, we present preliminary results giving evidence of the crucial role of iNOS for the anti-fibrotic properties of MSC.

## Materials and Methods

### Isolation and Culture of MSC

MSC from C57BL/6 wild-type-(WT)-mice or from IL6-, iNOS-knock-out C57BL/6J-mice, or IL1RA-knock-out BALB/c-mice (IL6^−/−^, IL1RA^−/−^, iNOS^−/−^-MSC, respectively) were isolated from bone-marrow (BM), as reported earlier (4, 5). BM was flushed out from long bones and the cell suspension was plated in DMEM supplemented with 10% fetal bovine serum (FBS) (PAA Laboratories GmbH, Austria), 2 mM glutamine, 100 U/ml penicillin, 100 mg/mL streptomycin (Lonza, France), and 2 ng/ml human bFGF (R&D Systems, France). Cells were passaged till obtaining homogeneity for mesenchymal marker expression and lack of hematopoietic markers as analyzed by flow cytometry. They were used between passages 10 and 15.

### HOCl Preparation

HOCl was generated extemporaneously by adding NaClO (9.6% as active chlorine) to KH_2_PO_4_ solution (100 mM, pH: 6.2), usually using a 1:100 ratio. The right amount of NaClO was adjusted so as to obtain the desired HOCl concentration, defined by the absorbance of the mixture at 292 nm (optical density between 0.7 and 0.9 read on a Nanodrop spectrophotometer, Thermoscientific). Stock solutions were stored at 4°C in the dark and NaClO was replaced every 3 weeks.

### Experimental Design and Animals

Six-week-old female BALB/c mice purchased from Janvier were housed and cared for according to the Laboratory Animal Care guidelines. Approval was obtained from the Regional Ethics Committee on Animal Experimentation (approval APAFIS#5351-2016050919079187) and the French Ministry for Education, Higher Education and Research. The mice had their backs shaved the day before the disease induction. Skin thickness was assessed with a caliper before disease induction and every week during the whole experiment by a blinded experimenter. As previously described, a total amount of 300 μL of freshly prepared HOCl was injected in two sites into the backs of the mice with a 29 G needle, 5 days a week for 6 weeks (8). Control mice received PBS in the same conditions. Treated-mice received an infusion of MSC (2.5x10^5^ cells conditioned in 100 μL PBS), in the tail vein at day 21. Groups of 7 to 10 mice were made for each condition (PBS-, HOCl-, and MSC-treated HOCl-mice). After 6 weeks and a 2-day recovery time without HOCl injections, all animals were sacrificed. Blood samples were collected and serum was recovered after centrifugation (1,500 g, 10 min) and stored at −20°C for ELISA. Skin biopsies (6 mm punches) were performed on the backs of the mice and lungs were removed and washed in PBS. Samples were stored at −80°C for RT-qPCR and collagen content determination or fixed in 4% formaldehyde for histopathological analysis.

### Histopathology

Skin samples were embedded in paraffin and 5μm thick sections were stained with Masson-trichrome. Histological slides were scanned using Nanozoomer (Hamamatsu).

### RT-qPCR Analysis

Skin samples were crushed in RLT-buffer and total RNA was extracted using the RNeasy mini-kit and Qiacube robotic workstation (Qiagen, France). One microgram of RNA was reverse-transcribed (M-MLV RT, Invitrogen, France). qPCR was performed on 20 ng cDNA using LightCycler480 SYBRGreenI Master-mix and real-time PCR instrument (Roche, France). Primers were designed using the applications Primer3 and BLAST as already described (2). Samples were normalized to mRNA expression of TATA binding protein *(Tbp*) housekeeping gene, and results provided either as relative expression to *tbp* using the formula 2^−ΔCt^ or as fold-change vs. PBS-mice using the formula 2^−ΔΔCt^.

### Collagen Content in Skin

Collagen content assay was based on the quantitative dye-binding Sircol method (Biocolor, Ireland). Skin biopsies taken from the site of injection were suspended in 2 mL of a 0.5 M acetic acid—pepsin (2.5 mg/mL) solution and dissociated using UltraTurrax (vWR, France). Collagen extraction was performed overnight at 4°C under stirring. The solution was then centrifuged at 12,000 g for 10 min and 20 μL of each sample were added to 1 mL of Syrius red reagent. Tubes were rocked at room temperature for 30 min and centrifuged at 12,000 g for 10 min. The supernatants were discarded and the tubes washed with 750 μL of ice-cold salt acid wash. After another 12,000 g centrifugation of 10 min, the collagen-dye pellets were resuspended in 1 ml of 0.5 M NaOH Alkali solution. Optical density (OD) was then read at 555 nm on a microplate reader (Varioskan Flash, Thermo scientific) vs. a standard range of bovine collagen type I concentrations (supplied as a sterile solution in 0.5 M acetic acid). Results were expressed as the collagen content in μg/mm^2^ of skin.

### Determination of Advanced Oxidation Protein Product (AOPP) Concentrations in Sera

AOPP concentration was measured by spectrophotometry as previously described (3). Twenty microliters of acetic acid was added to 200 μL of serum diluted 1:20 in PBS. In standard wells, 20 μL of acetic acid was added to 200 μL of chloramine-T solution (range from 0 to 1,000 μM) followed by 10 μL of 1.16 M potassium iodide. Absorbance was read at 340 nm on a microplate reader (Varioskan Flash) before and immediately after adding acetic acid and potassium iodide. AOPP concentration was expressed as chloramine-T equivalents (μM).

### Total Anti-oxidant Capacity of Serum

The total antioxidant capacity was determined on sera diluted 1:10, measuring the formation of the radical cation 2,29-azino-bis (3- ethylbenzthiazoline-6-sulfonic acid) using the Antioxidant Assay Kit (Cayman Chemical, Interchim, France). The absorbance was read at 750 nm on a microplate reader (Varioskan Flash) vs. a standard range of Trolox, and was expressed as mM Trolox equivalents.

### Determination of Glutathione (GSH) and Glutathione Disulfide (GSSG) Concentrations in Serum

At sacrifice, 100 μL of blood was collected with heparinized syringe, immediately mixed with 100-μL trichloro-acetic acid (10% in EDTA) and centrifuged at 10,000 g, at 4°C for 10 min allowing plasma recovery. Concentrations of GSH and its oxidized form GSSG were determined using ultraperformance liquid-chromatography–tandem-mass spectrometry (UPLC, Waters Acquity, Milford, USA).

### Statistical Analysis

Quantitative data were expressed as mean ± SEM. Data were compared using Mann–Whitney's test for non-parametric values or Student's *t*-test for parametric values as evaluated using the Shapiro–Wilk normality test. When analysis included more than two groups, one-way ANOVA was used. All statistical analyses were performed using Prism 6 GraphPad software for Mac OS (California, USA). A *P* < 0.05 was considered significant.

## Results

### iNOS Activity Is Required for MSC-Based Therapy of SSc

**Figure 1:**
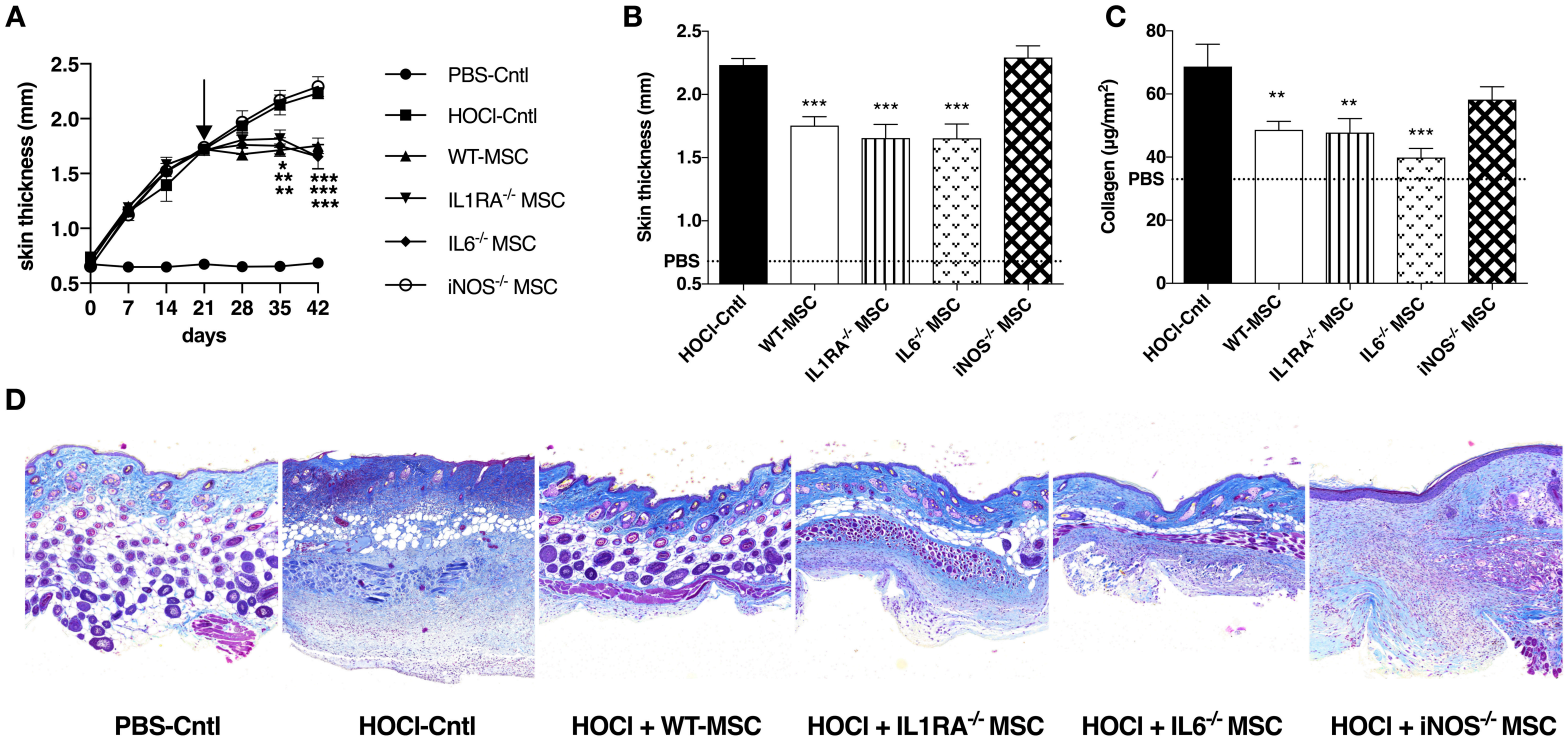
Comparative effects of WT MSC, IL1RA^−/−^, IL6^−/−^, and iNOS^−/−^ MSC in HOCl-SSc. **(A)** Skin thickness evolution (d0 to d42) from control PBS-mice, HOCl-mice and HOCl-mice treated with 2.5 × 10^5^ WT-, IL1RA^−/−^, IL6^−/−^, or iNOS^−/−^ MSC at d21. **(B)** Skin thickness at d42 in previously mentioned groups of mice (control PBS-mice are represented by a discontinued line). **(C)** Collagen content in skin samples from HOCl-mice and HOCl-mice treated with 2.5 × 10^5^ WT-, IL1RA^−/−^, IL6^−/−^, or iNOS^−/−^ MSC (mean level for control PBS-mice is represented by a discontinued line). **(D)** Representative skin sections at d42 (original magnification 10x; Masson Trichrome staining). *N* = 8 for PBS-mice, HOCl-mice, and IL6^−/−^ MSC-treated mice, *n* = 7 for IL1RA^−/−^ and iNOS^−/−^ MSC-treated mice. ^*^P < 0.05, ^**^P < 0.01, ^***^P < 0.001, data are presented as mean ± SEM.

In a first series of experiments, we compared the effects of MSC defective for IL1RA, IL6, and iNOS production with those of WT-MSC when injected during the course (d21) of HOCl-SSc. In this setting, we observed that disease progression was hampered in mice treated with WT, IL1RA^−/−^, or IL6^−/−^-MSC, indicating that neither IL1RA nor IL6 were involved in the therapeutic effect of MSC in this model (Figure 1A). Conversely, iNOS^−/−^-MSC did not affect the course of skin thickness, which followed the progression of non-treated HOCl-SSc mice. Concordantly, at d42, skin thickness was significantly lower in mice treated with WT, IL1RA^−/−^, or IL6^−/−^-MSC, compared with non-treated mice or iNOS^−/−^-MSC-treated mice; and no difference in skin thickness was found between the two latter groups (Figure 1B).

These clinical data were corroborated by the measurement of collagen content in skin, significantly lower in mice treated with WT, IL1RA^−/−^, or IL6^−/−^-MSC compared with HOCl-SSc mice, while no significant effect was noted for iNOS^−/−^-MSC-treated mice (Figure 1C). On histology, treatment with WT-MSC and to the same extent with IL1RA^−/−^ or IL6^−/−^-MSC reduced dermal collagen infiltration, while no reduction in collagen deposition was observed in iNOS^−/−^-MSC-treated mice (Figure 1D).

### iNOS^−/−^ MSC Show Preserved Anti-inflammatory and Remodeling Capacities

**Figure 2:**
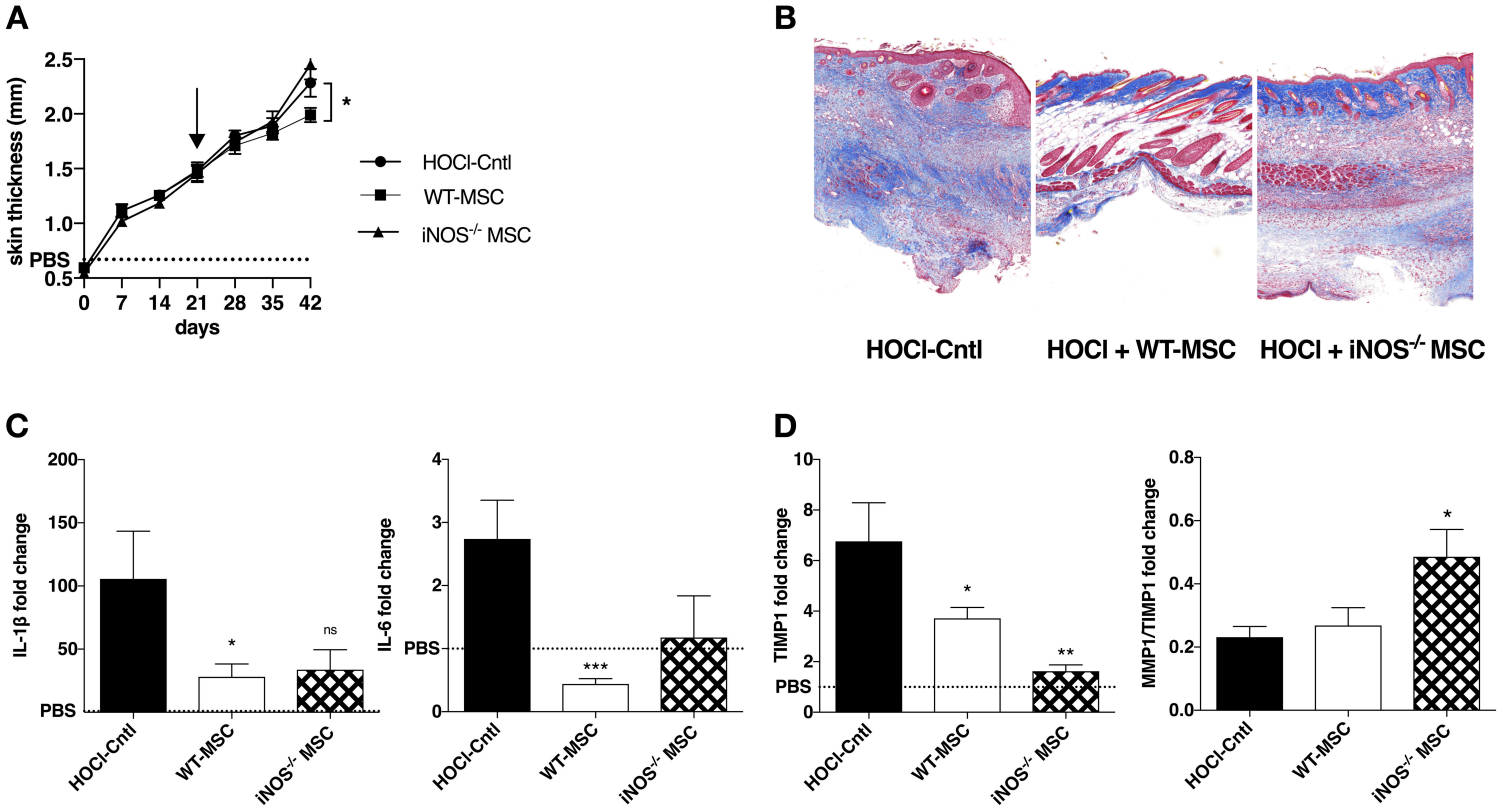
Effects of iNOS^−/−^ MSC on fibrosis, inflammation, and tissue remodeling in HOCl-SSc. **(A)** Skin thickness evolution (d0 to d42) from HOCl-mice and HOCl-mice treated with 2.5 × 10^5^ WT-, or iNOS^−/−^ MSCs at d21 (mean levels for control PBS-mice are represented by a discontinued line). **(B)** Representative skin sections at d42 (original magnification 10x; Masson Trichrome staining). **(C,D)** mRNA expression of *IL1*β*, IL6, MMP1*, and *MMP/TIMP1* at d42 in skin sections from HOCl-mice and HOCl-mice treated with 2.5 × 10^5^ WT-, or iNOS^−/−^ MSC. Results are given as fold-change vs. control PBS-mice normalized at 1.

Since IL1RA^−/−^ or IL6^−/−^-MSC exerted similar anti-fibrotic effects as WT_MSC, we next focused on iNOS^−/−^-MSC in a second series of experiments. We confirmed that iNOS^−/−^-MSC were unable to reduce skin thickening (Figure 2A) or collagen deposition (Figure 2B) during the induction of HOCl-SSc, compared with WT-MSC.

In order to decipher the underlying mechanisms, we then investigated the effect of MSC treatment on skin inflammation and tissue remodeling. We first noted that iNOS^−/−^-MSC were almost as efficient as WT-MSC in reducing the expression of IL1β and IL6, two main inflammatory cytokines that are found at high levels within the skin of HOCl-SSc mice (Figure 2C). Concerning their ability to improve ECM remodeling, we noted that mice treated with iNOS^−/−^-MSC disclosed reduced expression of tissue inhibitor of metalloprotease-1 (TIMP-1), and higher matrix metalloproteinase (MMP)1/TIMP1 ratio compared with HOCl-SSc mice, indicating enhanced remodeling capacity, similar to what is observed using WT-MSC (Figure 2D). Of note, concerning systemic involvement in HOCl-SSc, iNOS^−/−^-MSC were not able to reduce fibrotic markers such as collagen 1 and α-SMA and inflammatory cytokines (IL-1β and IL-6) in lung tissue, while WT-MSC had a positive impact on pulmonary fibrosis in this model (data not shown).

### iNOS^−/−^ MSCs Fail to Dampen HOCl-Induced Oxidative Stress

**Figure 3:**
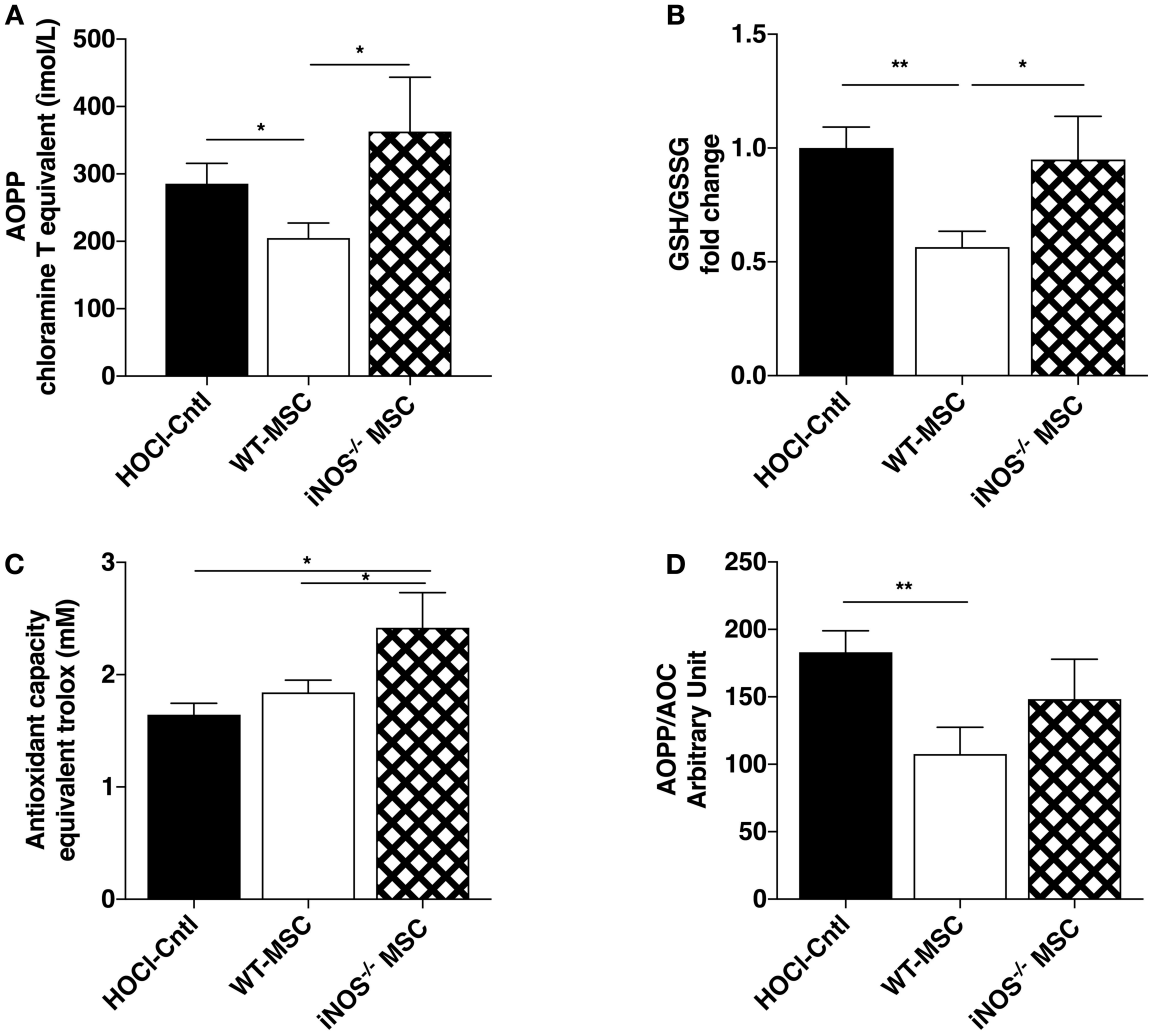
Effects of iNOS^−/−^ MSC on oxidative balance in HOCl-SSc. **(A)** Advanced Oxidation Protein Product (AOPP) concentrations in sera from HOCl-mice and HOCl-mice treated with 2.5 × 10^5^ WT-, or iNOS^−/−^ MSC. **(B)** Glutathione levels in sera from HOCl-mice and HOCl-mice treated with 2.5 × 10^5^ WT-, or iNOS^−/−^ MSC (GSH/GSSG ratio). **(C)** AOC and **(D)** AOPP/AOC serum levels in HOCl-mice and HOCl-mice treated with 2.5 × 10^5^ WT-, or iNOS^−/−^ MSCs. *N* = 7 HOCl-mice, *n* = 6 for HOCl-mice treated with 2.5 × 10^5^ WT-, or iNOS^−/−^ MSC. ^*^P < 0.05, ^**^P < 0.01, data are presented as mean ± SEM.

Since immunomodulatory and remodeling capacities of iNOS^−/−^-MSC seemed relatively preserved, we next looked at their effects on oxidative parameters in HOCl-SSc mice. Interestingly, we observed that iNOS^−/−^-MSC failed to reduce the levels of AOPP in serum compared with WT-MSC, but induced higher levels of gluthatione and enhanced anti-oxidant capacity (AOC) (Figures 3A–C). In the end, the overall oxidative balance represented by AOPP/AOC ratio remained high under iNOS^−/−^-MSC treatment whereas it significantly decreased under WT-MSC treatment (Figure 3D).

## Discussion

We previously demonstrated pleiotropic mechanism of MSC, acting through the abrogation of myofibroblastic activation and the reduction of tissue inflammation through potent immunosuppression, but also inducing tissue remodeling via metalloproteinase activation, and improving oxidative imbalance (2, 6, 7). These different mechanisms may depend on the pathological environment at the time of MSC infusion and argue for MSC adaptive capacities. This seems even more relevant in the setting of SSc, a heterogeneous and multifaceted disease.

In the present study, we aimed at deciphering possible mechanisms of MSC efficacy in HOCl-SSc and focused on their paracrine functions through molecules that were known to support their immunosuppressive capacities. Using MSC defective for these molecules, we observed that only iNOS seemed to be mandatory for the anti-fibrotic effects of MSC in HOCl-SSc. By contrast, IL1RA and IL6 were not involved in this function while they were required in previous *in vitro* and *in vivo* studies on other inflammatory models, such as collagen-induced-arthritis, a preclinical model for rheumatoid arthritis, another pathological condition where inflammatory cytokines such as IL6 play a critical role (4, 5).

Focusing on inflammation in mice treated with iNOS^−/−^-MSC, we noticed that there were slightly less capable of reducing cytokine production than WT-MSC. This seems consistent with the importance played by iNOS for the immunosuppressive function of MSC in literature (9). Another explanation for these results may lie in the fact that inflammation in HOCl-SSc mainly pertains to the onset of the fibrogenic process (before d21), a period of time where the role of MSC might be more devoted to immunosuppression (Frontiers Immunology, in revision).

Since we did not demonstrate a dramatic reduction of anti-inflammatory function using iNOS^−/−^-MSC, we looked for other mechanisms involved in the lack of therapeutic effect in murine SSc. We first turned to tissue remodeling, because this process seems of particular importance in the last 3 weeks of HOCl-SSc model (from d21 to d42), a phase characterized by less inflammation but strong collagen deposition in tissue. Interestingly, iNOS^−/−^-MSC improved some remodeling parameters, with higher levels of the MMP1/TIMP1 ratio in skin, as compared with those found in WT MSC treated-mice. The strong decrease of TIMP-1, one main inhibitor of metalloproteinases, under iNOS^−/−^ MSC treatment, suggested that iNOS is poorly involved in tissue remodeling.

Still, iNOS^−/−^ MSC failed to prevent collagen deposition in tissue, which led us to consider the impact on oxidative balance. Indeed, in HOCl-SSc as well as in human disease, the role of oxidative stress seems pivotal (10, 11). Notably, AOPP were reported to play a critical role in fibrosis and autoantibody formation both in human and mice (8, 12). Herein, we showed that iNOS^−/−^-MSC, unlike WT-MSC, were not able to reduce the levels of AOPP in serum. Even though anti-oxidant defenses (i.e., serum AOC and glutathione levels) were upregulated in iNOS^−/−^-MSC-treated mice, to even higher levels than in WT MSC-treated mice, the overall oxidative balance seemed unfavorable in these mice. In that sense, the strong upregulation in some parameters (i.e., glutathione or tissue remodeling enzymes MMP1/TIMP1), even overpassing what is observed using WT-MSC, might be a compensatory mechanism to counteract the persistent oxidative stress in these mice treated with defective iNOS^−/−^-MSC.

On the whole, through this concise report, we demonstrate the crucial role of iNOS in the therapeutic effects of MSC in the murine HOCl-SSc model resulting in a global anti-fibrotic impact. This is supported by another study where iNOS^−/−^-MSC failed to prevent tissue fibrosis in a model of liver cirrhosis (13). Conversely, another study reported that NO increased the anti-fibrotic properties of MSC in the same disease model (14).

Actually, NO plays a complex role in tissue remodeling and in oxidative stress regulation. On the one hand, the short-term production of NO by iNOS induces reactive oxygen species (ROS) formation such as peroxynitrites (15). On the other hand, NO is required for wound healing (16–18) and is also considered as an antioxidant (19, 20). Moreover, while prolonged iNOS blockade induces renal, heart or liver fibrosis in rodents (21–23), NO has shown proper antifibrotic roles, through the inhibition of myofibroblast activation, the abrogation of TGFβ pathway and the activation of MMP and hepatocyte growth factor (HGF), leading to less collagen deposition in other models (13, 20, 24). Interestingly, in the specific context of SSc, molecules up-regulating the NO pathway have been developed to treat pulmonary arterial hypertension in the clinics (25), and demonstrated anti-fibrotic effects in various preclinical models (26). This strengthens the interest of these preliminary results. Even if iNOS might not be the only mediator of importance in MSC therapeutic effects, this work underlines the role played by oxidative stress in SSc, and brings the perspective of enhancing MSC anti-oxidant activity to ameliorate their anti-fibrotic properties for future applications.

## Author Contributions

AM participated in the design of the study, acquisition, analysis and interpretation of data, manuscript redaction and final approval. PR, GF, TS, MM, and KT participated in acquisition and analysis of data, manuscript proofreading and final approval. J-PC and CJ participated in the design of the study, interpretation of data, manuscript preparation and final approval. DN and PG carried out the conception and design of the study, participated in analysis and interpretation of data, manuscript redaction and final approval.

### Conflict of Interest Statement

The authors declare that the research was conducted in the absence of any commercial or financial relationships that could be construed as a potential conflict of interest.
